# Cerclages after Femoral Osteotomy Are at Risk for Bacterial Colonization during Two-Stage Septic Total Hip Arthroplasty Revision

**DOI:** 10.7150/jbji.24819

**Published:** 2018-07-06

**Authors:** Viktor Janz, Georgi I. Wassilew, Carsten F. Perka, Michael Müller

**Affiliations:** Charité - Universitätsmedizin Berlin, Orthopaedic Department, Charitéplatz 1, 10117 Berlin, Germany

**Keywords:** Cerclage, Femoral Osteotomy, Two-Stage THA Exchange

## Abstract

**Aims:** In cases of a two-stage septic total hip arthroplasty (THA) exchange a femoral osteotomy with subsequent cerclage stabilization may be necessary to remove a well-fixed stem. This study aims to investigate the rate of bacterial colonization and risk of infection persistence associated with *in situ* cerclage hardware in two-stage septic THA exchange.

**Patients and Methods:** Twenty-three patients undergoing two-stage THA exchange between 2011 and 2016 were included in this retrospective cohort study. During the re-implantation procedure synovial fluid, periprosthetic tissue samples and sonicate fluid cultures (SFC) of the cerclage hardware were acquired.

**Results:** Seven of 23 (30%) cerclage-SFC produced a positive bacterial isolation. Six of the seven positive cerclage-SFC were acquired during THA re-implantation.

Two of the seven patients (29%) with a positive bacterial isolation from the cerclage hardware underwent a THA-revision for septic complications. The other five patients had their THA *in situ* at last follow-up.

**Conclusions:** Despite surgical debridement and antimicrobial therapy, a bacterial colonization of cerclage hardware occurs and poses a risk for infection persistence. All cerclage hardware should be removed or exchanged during THA reimplantation.

## Introduction

The main goal of a two stage hip revision in periprosthetic joint infection (PJI) is the elimination of all bacteria and biofilm. To eliminate the risk of residual bacterial reservoirs or biofilm a removal of all foreign material is preferred and the introduction of new hardware is avoided whenever possible 1, 2.

In cases of a well-fixed stem or extensive cementation an extended trochanteric osteotomy or an osseous window may be necessary during the explantation procedure also in septic cases 3-5. In these cases, an osteosynthesis using wire- or band-cerclages becomes necessary to fixate the osteotomy and restore the stability of the proximal femur 4, 5. However, this in turn means the introduction of new foreign material in a potentially septic environment.

Previous sonication studies were able to show that the bacterial biofilm in PJI encompasses the entire joint and all intraarticular components, independent of component type or material 6-8.

During septic two-stage total joint arthroplasty revision, this bacterial colonization also occurs to newly introduced foreign material, such as antibiotic-loaded polymethylmethacrylate (PMMA) spacers, after component explantation and surgical debridement 9-13. Despite the continuous antimicrobial therapy the bacterial colonization of PMMA-spacers, detected at the time of component reimplantation, has been shown to result in increased rates of future septic failure 11-13.

It is currently unclear if this bacterial colonization also occurs with newly implanted femoral cerclages after performance of a femoral osteotomy, to explant a well-fixed stem, in cases of septic two-staged THA-revision. Furthermore, it is unclear if a bacterial colonization of cerclage hardware also results in increased rates of future septic failure.

Hence, it was the goal of this study to investigate if a bacterial colonization of cerclage hardware occurs during two-stage septic THA revision and if this poses a risk for future septic failure.

## Patients and Methods

### Study design and patient population

Twenty-nine patients undergoing THA revision surgery between 2011 and 2016 were included in this retrospective cohort study. Approval from our institutional review board was obtained prior to commencement of this study. Inclusion criteria were removal of femoral cerclage hardware during septic two-stage THA revision. Exclusion criteria were a missing sonication of cerclage hardware, cerclage removal during the first-stage explantation procedure or cerclage removal for any other reason. Two patients were excluded because of a missing sonication of cerclage hardware and four patients were excluded due to incomplete microbiological sampling leaving 23 patients for evaluation. From these 23 patients four received a cerclage removal during a second round of irrigation and debridement (I&D) for PJI persistence and 19 patients received an explantation of the cerclage hardware during the second stage THA reimplantation.

The average patient age in our cohort was 74 years [49 - 91 years]. The average duration of the THAs *in situ* was 35 months and 14 of the 23 THAs had a failure within 24 months of implantation. The type of PJI was also assessed. There was one case of early PJI, 13 cases of delayed PJI and nine cases of late PJI 14. The average duration of the Girdlestone interval was 3,4 months [1,5-20 months].

The following samples were acquired intraoperatively: multiple periprosthetic tissue samples (minimum of five samples), periprosthetic membrane for histological samples, synovial fluid, as well as sonicate fluid cultures (SFC) of the explanted prosthetic parts (1^st^ stage) and cerclages (2^nd^ stage). Sonication was performed for three minutes using a BactoSonic 14.2 (Bandelin, Berlin, Germany) sonication unit with subsequent microbiological culture of the sonicate fluid in blood culture bottles and conventional agar plate cultures 15, 16. All microbiological samples, including sonicate fluid cultures, were cultured for 14 days to allow for the detection of fastidious species 17.

The presence of PJI was defined according to the following criteria: presence of intraarticular pus or a sinus tract, positive isolation of the same bacterial species in a minimum of two microbiological samples or a histological membrane indicative of infection 18, 19.

**Figure 1:**
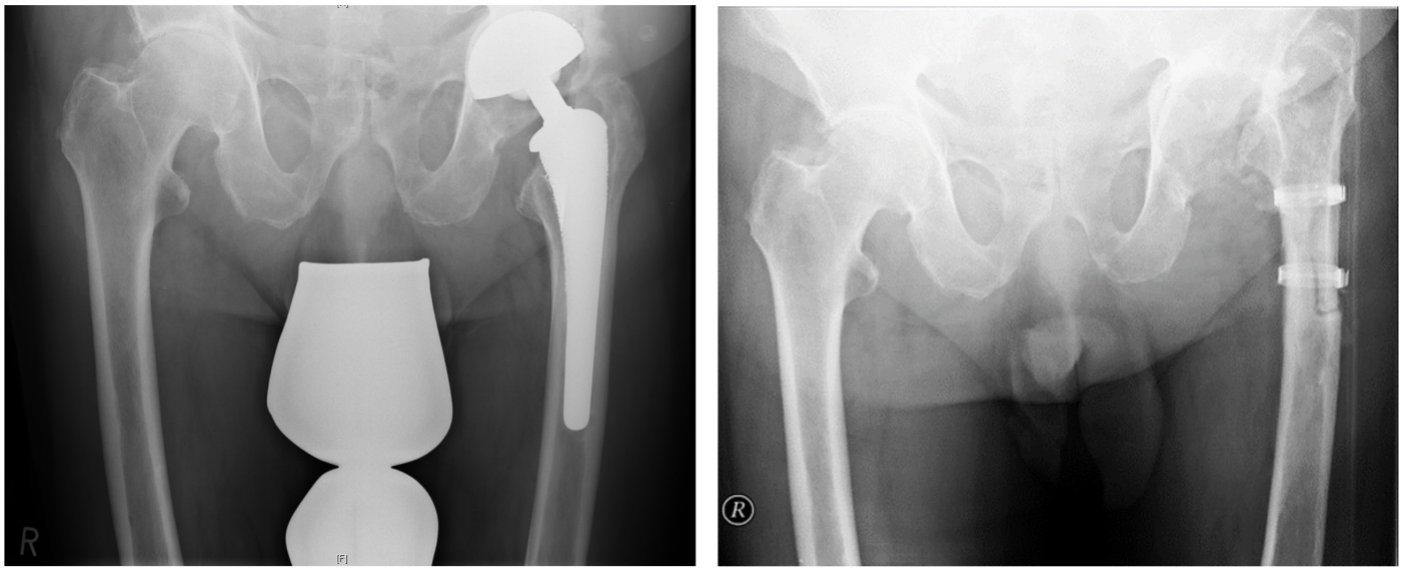
Pre- and postoperative AP pelvic radiograph of an infected THA. Explantation was performed by using an extended trochanteric osteotomy and re-fixation with two cerclages.

All of the 23 two-staged septic THA revisions included in this study were of cementless fixation and were treated at our department according to the following standardized protocol. After establishment of the diagnosis of PJI, the first stage explantation procedure consists of a complete explantation of all arthroplasty components and foreign materials to create a true Girdlestone resection arthroplasty. In all cases an extended trochanteric osteotomy or other femoral osteotomy was necessary to remove a well-fixed stem and cerclages were used to restore the stability of the proximal femur (Figure 1). During the explantation procedure the following samples were acquired: synovial fluid, multiple periprosthetic tissue samples, a sonication of the explanted arthroplasty components as well as a histological sample of the periprosthetic membrane. The grading of the periprosthetic membrane was performed according to the consensus classification of Morawietz et al 20. After the explantation procedure i.v. antibiotic therapy was initiated according to current international therapy guidelines 2, 19. In general, i.v. antibiotics were administered for the first two weeks, adjusted if necessary in accordance with the antibiotic sensibility, and continued orally for a minimum of four weeks 2. The specific oral antibiotic treatment is continued without interruption until the second stage reimplantation procedure^2^. No biofilm-active antibiotics were administered between the first and second stage. If the paraclinical serum infection parameters (CRP) were either continuously falling or within normal values after completion of a minimum of six weeks of antimicrobial therapy, then the THA-reimplantation was performed. If the paraclinical infection parameters were continuously elevated after completion of the antimicrobial therapy, or if there were clinical sign of infection persistence such as erythema or calor, this was interpreted as a persistence of PJI and a second round of I&D including an exchange of the existing ceclages was performed and an extended course of antimicrobial therapy for an additional six weeks was initiated.

From the 23 patients included in this study, 19 patients received an explantation of the cerclage hardware during the second-stage THA-reimplantation and four patients received an explantation of the cerclage hardware during a second I&D procedure for infection persistence.

The positive bacterial isolations from the individual microbiological methods were recorded at the time of THA-explantation and THA-reimplantation (Table 1). The further clinical outcome after THA-reimplantation was also recorded for all patients and classified as *in situ*, I&D with or without bearing exchange and THA-revision or THA-explantation.

All statistical calculations were performed using SPSS 15 (Statistical Package for Social Sciences, Inc., Chicago, IL, USA). The statistical significances for risk of THA-revision between the patients with positive cerclage-SFC and patients with negative cerclage-SFC was calculated using Χ^2^ test and the cut off for statistical significance was defined as p = 0,05.

## Results

In our entire patient cohort seven of 23 (30%) cerclage-SFC produced a positive bacterial isolation. Six positive bacterial isolations were from cerclage-SFC during THA-reimplantation and one positive isolation was from a cerclage-SFC during a second I&D procedure. Table 1 displays the results of the intraoperative microbiological samples for the 19 patients undergoing THA re-implantation. Six of these 19 patients (32%) produced a positive bacterial isolation by cerclage-SFC. In accordance with our criteria for THA re-implantation, none of these patients presented any preoperative clinical or laboratory signs of infection persistence (erythema, calor, persistent pain or elevated serum CRP) prior to second-stage surgery.

To investigate the clinical relevance of a positive cerclage-SFC, the further clinical course and survival of the 19 cases, with a sonication of cerclage hardware from the second-stage THA reimplantation, were recorded. Six of theses 19 patients, showed a positive bacterial growth in the cerclage-SFC. Only one of these six patients fulfilled the PJI criteria, due to a positive histology at THA reimplantation. From these six patients four patients underwent revision surgery (66%). Two patients received an additional I&D, one patient received a THA explantation and one patient a cup exchange. Thirteen of 19 patients showed no growth in the cerclage-SFC from the second-stage THA reimplantation. From these 13 patients only a single patient fulfilled the PJI criteria and four patients underwent revision surgery (31%). Three received an I&D and one patient received a stem exchange. The group with positive cerclage-SFC had a higher revision rate than the group with negative cerclage-SFC, 31% vs. 66%, although this difference was not significant (p= 0,14).

## Discussion

Our data shows that femoral cerclages, implanted during the explantation procedure, represent a risk factor for bacterial colonization and persistence during septic two stage THA exchange. This means colonization also occurs on femoral cerclages introduced after femoral osteotomy during THA explantation for PJI, despite the surgical debridement and postoperative antimicrobial therapy. While previous studies have been able to demonstrate that the bacterial colonization in PJI is present on PMMA-spacers during two-stage septic revision surgery, this study is the first to show, that this colonization is also present on cerclage hardware 9-13.

Currently, there are no recommendations in the literature regarding the management of *in situ* cerclage hardware in the setting of a two-staged septic THA exchange. If a femoral osteotomy becomes necessary to explant a well-fixed stem a re-fixation of the osteotomy through sutures, wire-, cable- or band-cerclages becomes necessary. In turn, this results in the dilemma of introducing new hardware into a septic, albeit a freshly debrided, situs.

One possible solution is the utilization of alternative fixation materials, such as sutures, for the refixation of the femoral osteotomy 21. This method was first described with the intention of reducing the mechanical complications of wire or cable cerclages, such as disruption of the osteotomy's blood supply or avoiding metal on metal contact. However, it remains unclear if the utilization of sutures might reduce the bacterial colonization present on foreign body hardware during the first- and second-stage interval 21. Previous sonication studies have shown that there is an even higher bacterial adherence to synthetic surfaces, such as PE and possibly sutures, in comparison to metal or ceramic surfaces 7.

The major limitation of this study is the small sample size, despite the long inclusion period. This is due to the fact that the combination of a femoral osteotomy and a two-stage THA exchange is a relatively rare event, even at a large tertiary care center. Due to the small sample size, the investigation of patient specific risk factors for bacterial colonization of cerclage hardware or infection persistence is not possible, due to a lack of statistical power. Additionally, a longer follow up period might reveal an even higher rate of septic failure for the cases with a positive SFC of cerclage hardware.

A second limitation of this study is indebted to the fact that no spacers were used and a true Girdlestone resection arthroplasty was performed. We do not employ PMMA-spacers due to the high rate of mechanical complications, such as spacer breakage or dislocation and the contraindicated use of spacers in the presence of severe acetabular osseous defects 22-24. However, the majority of the published results in the current literature include PMMA-spacer making a direct comparison with our results difficult 9-13. Although a theoretical argument could be made, that the presence of an antibiotic-eluting spacer could decrease the rate of bacterial colonization of the *in situ* cerclage hardware, there is no evidence to support this assumption. Sonication of retrieved of antibiotic-eluting spacers have shown that a bacterial colonization of the spacer surface occurs and that this colonization is highly predictive of future septic failure 9, 11-13, 25.

The interpretation of the bacterial species isolated from the cerclage hardware is also encumbered by the small sample size. The isolation of a bacterial species from the cerclage hardware differing from the bacterial species isolated during the first-stage explantation procedure is a common problem, reported in all sonication studies investigating PMMA-spacers 9-13, 25. This phenomenon of isolating a differing bacterial species between the first- and second-stage surgery, allows several possible interpretations. Firstly, polymicrobial infections are reported in up to 30% of all cases of PJI and the detection rate of polymicrobial infections through sonication is superior to that of the conventional microbiological methods 26. Secondly, it is possible that the species isolated from the cerclage hardware were selected through the continuous antimicrobial therapy given from the THA-explantation until THA-reimplantation 27. The selection of small colony variants is known to develop under antimicrobial therapy, pose a risk factor for infection persistence and have been isolated in up to 34% of PJI-cases 27-31. The other two possible explanations are that the additional species represent either an intraoperative contamination during THA reimplantation or a contamination of the microbiological sample, either during intraoperative acquisition, sample processing or the culture process.

The results of this study exemplify the importance of removing all foreign material, including cerclage hardware, in cases of PJI, since all foreign material poses a potential reservoir for residual bacteria 9, 11, 13, 25. While this study is the first to report the risk of bacterial colonization associated with retained cerclage hardware in two stage THA exchange, this has already been shown for two stage septic exchanges with spacers in knees and hips 9, 11, 13. Also, this study is the first to demonstrate the diagnostic benefits of a sonication of removed cerclage hardware.

In conclusion, while femoral cerclages are necessary to restore the stability of the proximal femur after prior osteotomy, they also pose the risk of bacterial colonization. Due to this bacterial colonization, cerclage hardware should be removed or exchanged during second- stage reimplantation and in cases of unsure infection control, the surgeon's threshold for performing a second debridement and removal of all cerclage hardware should be very low.

## Figures and Tables

**Table 1:** Overview over the microbiological culture results of the first-stage explantation and second-stage reimplantation procedure for patients undergoing two-stage septic THA exchange.

Case Nr.	Explantation procedure (first-stage)	Synovial aspiration or peri-prosthetic tissue (second-stage)	Cerclage sonication (second-stage)
1	Staph. epidermidis	*without growth*	*without growth*
2	Staph. sanguinis	Staph. epidermidis	Staph haemolyticus
4	Staph. aureus & Enterococcus faecalis	Staph. epidermidis	*without growth*
6	Prop. acnes	*without growth*	*without growth*
7	Staph. epidermidis	*without growth*	*without growth*
9	Staph. epidermidis	*without growth*	Micrococcus luteus
10	Enterococcus faecalis	*without growth*	Staph. hominis
11	*without growth*	*without growth*	*without growth*
12	Finegoldia magna	*without growth*	*without growth*
13	Staph. aureus	*without growth*	*without growth*
14	Staph. epidermidis + Staph. haemolyticus	*without growth*	Prop. acnes
15	Staph. epidermidis	*without growth*	*without growth*
17	Propionibacterium acnes	*without growth*	*without growth*
18	*without growth*	*without growth*	Staph. cohnii
19	*without growth*	*without growth*	*without growth*
20	Finegoldia magna	*without growth*	*without growth*
21	Escherichia coli	Staph. epidermidis	*without growth*
22	Staph. lugdunensis & Staph. epidermidis	*without growth*	Staph. epidermidis
23	MRSA	*without growth*	*without growth*

## References

[1] Frommelt L (2009). Diagnosis and treatment of foreign-body-associated infection in orthopaedic surgery. Der Orthopade.

[2] Zimmerli W, Trampuz A, Ochsner PE (2004). Prosthetic-joint infections. The New England journal of medicine.

[3] de Menezes DF, Le Beguec P, Sieber HP, Goldschild M (2012). Stem and osteotomy length are critical for success of the transfemoral approach and cementless stem revision. Clinical orthopaedics and related research.

[4] Fink B, Oremek D (2016). The Transfemoral Approach for Removal of Well-Fixed Femoral Stems in 2-Stage Septic Hip Revision. The Journal of arthroplasty.

[5] Fink B, Grossmann A (2007). Modified transfemoral approach to revision arthroplasty with uncemented modular revision stems. Operative Orthopadie und Traumatologie.

[6] Holinka J, Pilz M, Hirschl AM, Graninger W, Windhager R, Presterl E (2012). Differential bacterial load on components of total knee prosthesis in patients with prosthetic joint infection. The International journal of artificial organs.

[7] Lass R, Giurea A, Kubista B, Hirschl AM, Graninger W, Presterl E (2014). Bacterial adherence to different components of total hip prosthesis in patients with prosthetic joint infection. International orthopaedics.

[8] Gomez-Barrena E, Esteban J, Medel F, Molina-Manso D, Ortiz-Perez A, Cordero-Ampuero J (2012). Bacterial adherence to separated modular components in joint prosthesis: a clinical study. Journal of orthopaedic research: official publication of the Orthopaedic Research Society.

[9] Mariconda M, Ascione T, Balato G, Rotondo R, Smeraglia F, Costa GG (2013). Sonication of antibiotic-loaded cement spacers in a two-stage revision protocol for infected joint arthroplasty. BMC musculoskeletal disorders.

[10] Bereza P, Ekiel A, Augusciak-Duma A, Aptekorz M, Wilk I, Kusz D (2016). Comparison of cultures and 16S rRNA sequencing for identification of bacteria in two-stage revision arthroplasties: preliminary report. BMC musculoskeletal disorders.

[11] Nelson CL, Jones RB, Wingert NC, Foltzer M, Bowen TR (2014). Sonication of antibiotic spacers predicts failure during two-stage revision for prosthetic knee and hip infections. Clinical orthopaedics and related research.

[12] Sorli L, Puig L, Torres-Claramunt R, Gonzalez A, Alier A, Knobel H (2012). The relationship between microbiology results in the second of a two-stage exchange procedure using cement spacers and the outcome after revision total joint replacement for infection: the use of sonication to aid bacteriological analysis. The Journal of bone and joint surgery British volume.

[13] Esteban J, Gadea I, Perez-Jorge C, Sandoval E, Garcia-Canete J, Fernandez-Roblas R (2016). Diagnosis of spacer-associated infection using quantitative cultures from sonicated antibiotics-loaded spacers: implications for the clinical outcome. European journal of clinical microbiology & infectious diseases: official publication of the European Society of Clinical Microbiology.

[14] Tande AJ, Patel R (2014). Prosthetic joint infection. Clinical microbiology reviews.

[15] Shen H, Tang J, Wang Q, Jiang Y, Zhang X (2015). Sonication of explanted prosthesis combined with incubation in BD bactec bottles for pathogen-based diagnosis of prosthetic joint infection. Journal of clinical microbiology.

[16] Portillo ME, Salvado M, Trampuz A, Siverio A, Alier A, Sorli L (2015). Improved diagnosis of orthopedic implant-associated infection by inoculation of sonication fluid into blood culture bottles. Journal of clinical microbiology.

[17] Schafer P, Fink B, Sandow D, Margull A, Berger I, Frommelt L (2008). Prolonged bacterial culture to identify late periprosthetic joint infection: a promising strategy. Clinical infectious diseases: an official publication of the Infectious Diseases Society of America.

[18] Berbari EF, Hanssen AD, Duffy MC, Steckelberg JM, Ilstrup DM, Harmsen WS (1998). Risk factors for prosthetic joint infection: case-control study. Clinical infectious diseases: an official publication of the Infectious Diseases Society of America.

[19] Osmon DR, Berbari EF, Berendt AR, Lew D, Zimmerli W, Steckelberg JM (2013). Diagnosis and management of prosthetic joint infection: clinical practice guidelines by the Infectious Diseases Society of America. Clinical infectious diseases: an official publication of the Infectious Diseases Society of America.

[20] Morawietz L, Classen RA, Schroder JH, Dynybil C, Perka C, Skwara A (2006). Proposal for a histopathological consensus classification of the periprosthetic interface membrane. Journal of clinical pathology.

[21] Kuruvalli RR, Landsmeer R, Debnath UK, Suresh SP, Thomas TL (2008). A new technique to reattach an extended trochanteric osteotomy in revision THA using suture cord. Clinical orthopaedics and related research.

[22] Neumann DR, Hofstaedter T, List C, Dorn U (2012). Two-stage cementless revision of late total hip arthroplasty infection using a premanufactured spacer. The Journal of arthroplasty.

[23] Faschingbauer M, Reichel H, Bieger R, Kappe T (2015). Mechanical complications with one hundred and thirty eight (antibiotic-laden) cement spacers in the treatment of periprosthetic infection after total hip arthroplasty. International orthopaedics.

[24] Disch AC, Matziolis G, Perka C (2007). Two-stage operative strategy without local antibiotic treatment for infected hip arthroplasty: clinical and radiological outcome. Archives of orthopaedic and trauma surgery.

[25] Cabo J, Euba G, Saborido A, Gonzalez-Panisello M, Dominguez MA, Agullo JL (2011). Clinical outcome and microbiological findings using antibiotic-loaded spacers in two-stage revision of prosthetic joint infections. The Journal of infection.

[26] Janz V, Wassilew GI, Kribus M, Trampuz A, Perka C (2015). Improved identification of polymicrobial infection in total knee arthroplasty through sonicate fluid cultures. Archives of orthopaedic and trauma surgery.

[27] Proctor RA, von Eiff C, Kahl BC, Becker K, McNamara P, Herrmann M (2006). Small colony variants: a pathogenic form of bacteria that facilitates persistent and recurrent infections. Nature reviews Microbiology.

[28] Tande AJ, Osmon DR, Greenwood-Quaintance KE, Mabry TM, Hanssen AD, Patel R (2014). Clinical characteristics and outcomes of prosthetic joint infection caused by small colony variant staphylococci. mBio.

[29] Bogut A, Niedzwiadek J, Koziol-Montewka M, Strzelec-Nowak D, Blacha J, Mazurkiewicz T (2014). Characterization of Staphylococcus epidermidis and Staphyloccocus warneri small-colony variants associated with prosthetic-joint infections. Journal of medical microbiology.

[30] Maduka-Ezeh AN, Greenwood-Quaintance KE, Karau MJ, Berbari EF, Osmon DR, Hanssen AD (2012). Antimicrobial susceptibility and biofilm formation of Staphylococcus epidermidis small colony variants associated with prosthetic joint infection. Diagnostic microbiology and infectious disease.

[31] Schmolders J, Hischebeth GT, Friedrich MJ, Randau TM, Wimmer MD, Kohlhof H (2014). Evidence of MRSE on a gentamicin and vancomycin impregnated polymethyl-methacrylate (PMMA) bone cement spacer after two-stage exchange arthroplasty due to periprosthetic joint infection of the knee. BMC infectious diseases.

